# A Rare Dual Pathology in Refractory Chronic Rhinosinusitis: Maxillary Ectopic Tooth With Fungal Ball

**DOI:** 10.1155/crot/9522364

**Published:** 2026-04-23

**Authors:** Yu-Ru Lin, Ying-Chou Lu, Yen-Ting Lu

**Affiliations:** ^1^ School of Medicine, Chung Shan Medical University, Taichung, Taiwan, csmu.edu.tw; ^2^ Department of Otolaryngology, St. Martin De Porras Hospital, Chiayi, Taiwan; ^3^ Department of Otolaryngology, Chung Shan Medical University Hospital, Taichung, Taiwan, csh.org.tw

**Keywords:** ectopic tooth, functional endoscopic sinus surgery (FESS), fungal ball, recurrent rhinosinusitis, unilateral rhinosinusitis

## Abstract

Ectopic teeth in the maxillary sinus are an uncommon finding, and when symptomatic, often present as chronic rhinosinusitis. However, concurrent presentation with a fungal ball is exceptionally rare and scarcely documented in the literature. We report a unique case of a 39‐year‐old woman with recurrent maxillary sinusitis, ultimately diagnosed with coexisting ectopic tooth and fungal ball in the left maxillary sinus. The patient underwent successful endoscopic sinus surgery for simultaneous removal of both lesions. Postoperatively, she experienced complete resolution of symptoms without complications. This case highlights the diagnostic importance of imaging in refractory rhinosinusitis and demonstrates that endoscopic management is an effective and minimally invasive approach for this rare dual pathology.

## 1. Introduction

Teeth growing in the paranasal sinuses are a rare phenomenon. Among maxillary, ethmoid, sphenoid, and frontal sinuses, ectopic teeth emerging in the maxillary sinuses are most frequently reported, and the tooth type can vary from dental fragments such as tooth roots to full‐grown teeth [1–3].

Case reports of ectopic teeth growing in maxillary sinuses are tremendously scarce [2–6]. To date, only a limited number of cases have been reported in the literature, with few cases of ectopic tooth existing with a fungal ball documented in English‐language journals [7].

Hence, this study presents a case of a 39‐year‐old female who has a left maxillary ectopic tooth accompanied by a fungal ball. With a history of left fungal rhinosinusitis, our patient was previously treated with functional endoscopic sinus surgery (FESS) in 2015. Nonallergic fungal rhinosinusitis treated with FESS has a high success rate and recurrence is rarely seen [8]. In this article, we will explore the causes behind her recurrence of fungal rhinosinusitis, its connection to an ectopic tooth, and the rationale behind our chosen treatment approach.

## 2. Case Presentation

A 39‐year‐old woman, with a history of left fungal rhinosinusitis status post FESS in 2015 at another institution; however, prior operative records and imaging studies were not available for review. The patient had no history of trauma, dental pathology, or developmental disorders. She developed recurrent left‐sided nasal obstruction and thick rhinorrhea 8 years after the first surgery, with symptoms progressing gradually. The patient did not exhibit symptoms of a toothache, and a dental examination revealed no missing teeth, cavities, or gingivitis by the dentist. She then sought help at our outpatient department in February 2023.

**FIGURE 1:**
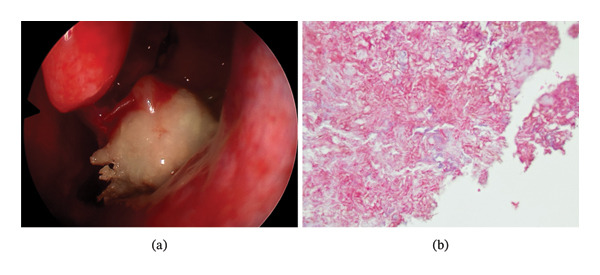
(a). Intraoperative endoscopic view showing a round, well‐demarcated fungal ball located in the left maxillary sinus and osteomeatal complex during functional endoscopic sinus surgery (FESS). (b). Histopathological image (H&E stain, 200 × magnification) showing inflamed respiratory epithelium with septate fungal hyphae consistent with fungal ball.

**FIGURE 2:**
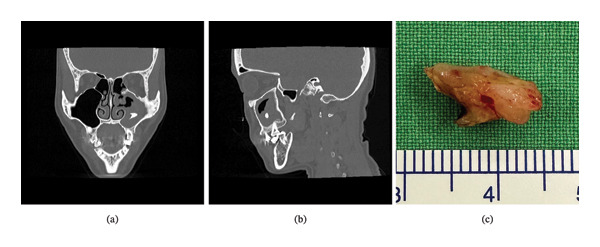
(a). Coronal computed tomography (CT) scan revealing left maxillary sinus opacification with heterogeneous density and an ectopic tooth—a well‐defined, radiopaque structure within the sinus cavity. (b). Parasagittal computed tomography (CT) scan showing no periapical lesion or involvement of adjacent normal teeth, excluding conventional odontogenic sinusitis. (c). Gross specimen of the ectopic tooth removed from the maxillary sinus, demonstrating a white, smooth‐surfaced structure measuring 1.3 × 0.7 × 0.7 cm^3^.

Diagnostic nasal endoscopy demonstrated a firm, dark‐colored mass with thick, peanut butter–like material and purulence obstructing the left osteomeatal complex (Figure 1(a)). Coronal computed tomography revealed near‐complete opacification of the left maxillary sinus with heterogeneous internal density. A well‐defined, radiopaque object consistent with an odontogenic structure was observed within the sinus cavity (Figure 2(a)). Parasagittal CT images demonstrated no periapical pathology or involvement of adjacent normal dentition, thereby excluding conventional odontogenic sinusitis (Figure 2(b)).

Given the persistent symptoms and imaging findings, FESS was performed. Intraoperatively, after a standard middle meatal antrostomy, the widened maxillary ostium allowed visualization of a compact fungal concretion and a white, smooth‐surfaced mass over the left maxillary sinus and adjacent OMC (Figure 2(c)). The ectopic tooth is neither embedded in the sinus mucosa nor attached to the bony wall.

Histopathological examination confirmed the presence of inflamed respiratory epithelium, stromal edema, vascular congestion, and marked eosinophilic infiltration (> 10 eosinophils per high‐power field). Numerous septate fungal hyphae with acute‐angle branching—features consistent with *Aspergillus* spp.—were identified (Figure 1(b)). The excised hard mass was confirmed histologically to contain enamel, dentin, and cementum, verifying the diagnosis of an ectopic tooth.

Based on the pathological findings and clinical history, the patient was diagnosed with the coexistence of an ectopic tooth and recurrent fungal rhinosinusitis in the maxillary sinus. Postoperatively, she tolerated the procedure well, and her rhinosinusitis resolved. No recurrence of symptoms was observed during the 1‐year follow‐up.

## 3. Discussion

The occurrence of teeth within the paranasal sinuses is an infrequent phenomenon [9]. Among all the sinonasal cavities, ectopic teeth are most frequently reported in the maxillary sinuses [1, 2, 4–6]. The types of teeth identified can vary, ranging from dental fragments, such as tooth roots, to fully developed teeth [1]. The etiology of ectopic teeth remains unclear. Various factors, including trauma, dentigerous or neoplastic cysts, inflammation, anatomical crowding, growth obstruction or retention, radiation exposure, cleft palate, hereditary factors, or iatrogenic incidents, have been implicated in the development of ectopic teeth [10]. Treatment of symptomatic ectopic teeth is generally surgical extraction [9, 11]. Based on the route of access, treatments range from external approaches like the de‐gloving procedure to Caldwell–Luc (CWL) operation or FESS [1, 9–11].

Fungal sinusitis can be classified into noninvasive and invasive types [12, 13]. Noninvasive fungal rhinosinusitis, such as a fungal ball or allergic fungal rhinosinusitis, tends to present in immune‐competent patients [12]. Unlike allergic fungal rhinosinusitis, a hypersensitivity response against the fungus, a fungal ball is a combination of fungal fragments, inflammatory infiltrates, cellular debris, and mucus forming a sphere‐like mass, minimizing the immune response by the host [12, 14]. On CT images, intrasinus hyperintensity, changes in the sinus wall, and sometimes calcification signals can be noticed [13].

Surgical treatment is the main policy for maxillary fungal balls with a low recurrence rate, usually done with internal approaches (e.g., CWL or FESS), but endoscopic methods are preferred [9]. In a meta‐analytic study conducted by Fadda and Allevi [8], endoscopic sinus surgery is stated as the only treatment for paranasal sinus fungus ball, with a treatment mean success rate of 98.4% (95% confidence interval [CI] 97.4%–99.3%), and recurrence is rarely seen.

As noted earlier, ectopic teeth occurring in the maxillary sinus are rare findings [2–6]. A recent report published in 2025 described fungal sinusitis associated with an ectopic tooth [3], and a Korean case also reported the coexistence of an ectopic tooth and a fungal ball in the maxillary sinus [7]. These reports indicate that such presentations have been described although documentation in the English‐language medical literature remains relatively limited.

In our case, histopathological examination confirmed a noninvasive fungal ball. The clinical significance of this case lies not only in the coexistence of an ectopic tooth and a maxillary sinus fungal ball but also in its recurrent presentation. In general, a noninvasive fungal ball is associated with a low recurrence rate following complete surgical treatment [8]. In the previously reported cases involving concomitant ectopic tooth and fungal ball, both lesions were identified and managed during the initial operation, with favorable postoperative outcomes. By contrast, in our patient, the ectopic tooth was not recognized at the time of the previous surgery. Although the fungal lesion was removed, the persistent ectopic tooth may have remained as an underlying odontogenic nidus, contributing to recurrent fungal rhinosinusitis and the need for revision surgery. After complete surgical removal of both the fungal ball and the ectopic tooth, no recurrence was observed during a 3‐year follow‐up. This case, therefore, adds to the limited literature on this rare entity and underscores the importance of carefully evaluating for an ectopic tooth in patients with maxillary fungal ball, particularly in recurrent cases.

Based on the multiple factors contributing to a fungal ball, it is possible that the ectopic tooth in the maxillary sinus, a dental factor, blocked normal sinus drainage and created an ideal anaerobic environment for fungal ball formation, which also concomitantly became the nidus of inflammation. Unresolved symptoms following previous FESS treatment could, therefore, be attributed to this previously unidentified ectopic tooth, and thus low recurrence of fungal rhinosinusitis after endoscopic procedures could not be applied.

Surgery is the best solution for both the ectopic tooth and the fungal ball, and an internal approach would be preferred with its smaller wound and shorter recovery phase. While the CWL procedure remains the most traditional approach to ectopic teeth in maxillary sinuses, there is a potential risk of damaging the infraorbital or superior anterior alveolar nerve [1, 8, 10]. On the other hand, the endoscopic approach is not only the treatment of choice for paranasal fungal balls, and it also boasts a lower rate of complication, surgical trauma, and postoperative rhinosinusitis [15]. Moreover, under FESS, other associated sinonasal pathologies can be simultaneously treated [15]. Thus, FESS was opted to treat our patient. In contrast to her last surgical treatment in 2015, the fungal ball was extracted along with the ectopic tooth during this visit, after which symptoms of our patient improved, and no recurrence or complications during her follow‐up visits.

## 4. Conclusion

Ectopic teeth of the maxillary sinus are uncommonly encountered, and their coexistence with a fungal ball is exceedingly uncommon, with minimal documentation in current literature. This case illustrates a patient with recurrent maxillary fungal rhinosinusitis, unresponsive to prior treatment, in whom an ectopic tooth was later identified as a contributing factor. Combined endoscopic removal of the fungal ball and ectopic tooth resulted in complete symptom resolution without complications. This underscores the importance of considering odontogenic etiologies in unilateral, refractory maxillary sinusitis and highlights the careful evaluation of radiologic findings in such cases.

## Author Contributions

Yu‐Ru Lin was responsible for data curation, methodology, project administration, and drafting the original manuscript. Ying‐Chou Lu contributed to investigation, project oversight, supervision, and critical revision of the manuscript. Yen‐Ting Lu contributed to conceptualization, investigation, methodology, project administration, supervision, drafting of the original manuscript, and critical review and editing.

## Funding

No funding was obtained for this study.

## Disclosure

This case report was reviewed and approved by the Institutional Review Board of St. Martin De Porres Hospital, Chiayi, Taiwan (IRB no. 25B‐008).

## Conflicts of Interest

The authors declare no conflicts of interest.

## Data Availability

The data that support the findings of this study are available from the corresponding author upon reasonable request.
